# Application of the research electronic data capture (REDCap) system in a low- and middle income country– experiences, lessons, and challenges

**DOI:** 10.1007/s12553-021-00600-3

**Published:** 2021-09-25

**Authors:** O. Odukoya, D. Nenrot, H. Adelabu, N. Katam, E. Christian, J. Holl, A. Okonkwo, M. Kocherginsky, K.-Y. Kim, S. Akanmu, F. B. Abdulkareem, R. Anorlu, J. Musa, O. Lesi, C. Hawkins, O. Okeke, W. L. Adeyemo, S. Sagay, R. Murphy, L. Hou, F. T. Ogunsola, F. H. Wehbe

**Affiliations:** 1 Department of Community Health and Primary Care, College of Medicine, University of Lagos, Lagos State, Nigeria; 2 College of Health Sciences, University of Jos, Plateau state, Nigeria; 3 AIDS Prevention Initiative of Nigeria, Lagos University Teaching Hospital, Lagos State, Nigeria; 4 Robert H. Lurie Comprehensive Cancer Center, Northwestern University, Chicago, IL, USA; 5 Havey Institute for Global Health, Feinberg School of Medicine, Northwestern University, Chicago, IL, USA; 6 Department of Neurology and Center for Healthcare Delivery Science and Innovation, Biological Sciences Division, University of Chicago, Chicago, IL, USA; 7 Research Management Office, College of Medicine, University of Lagos, Lagos, Nigeria; 8 Department of Preventive Medicine, Division of Biostatistics, Feinberg School of Medicine, Northwestern University, Chicago, IL, USA; 9 Department of Anatomic & Molecular Pathology, College of Medicine, University of Lagos, Lagos State, Nigeria; 10 Department of Obstetrics and Gynaecology, College of Medicine, University of Lagos, Lagos State, Nigeria; 11 Department of Medicine, College of Medicine, University of Lagos, Lagos State, Nigeria; 12 Division of Infectious Diseases, Department of Medicine, Feinberg School of Medicine, Northwestern University, Chicago, IL, USA; 13 Department of Maxillofacial Surgery, College of Medicine, University of Lagos, Lagos State, Nigeria; 14 Department of Medical Microbiology and Parasitology, College of Medicine, University of Lagos, Lagos State, Nigeria

**Keywords:** Redcap, Data capture, Research collaboration, LMIC

## Abstract

The challenges of reliably collecting, storing, organizing, and analyzing research data are critical in low- and middle-income countries (LMICs), particularly in Sub-Saharan Africa where several healthcare and biomedical research organizations have limited data infrastructure. The Research Electronic Data Capture (REDCap) System has been widely used by many institutions and hospitals in the USA for data collection, entry, and management and could help solve this problem. This study reports on the experiences, challenges, and lessons learned from establishing and applying REDCap for a large US-Nigeria research partnership that includes two sites in Nigeria, (the College of Medicine of the University of Lagos (CMUL) and Jos University Teaching Hospital (JUTH)) and Northwestern University (NU) in Chicago, Illinois in the United States. The largest challenges to this implementation were significant technical obstacles: the lack of REDCap-trained personnel, transient electrical power supply, and slow/intermittent internet connectivity. However, asynchronous communication and on-site hands-on collaboration between the Nigerian sites and NU led to the successful installation and configuration of REDCap to meet the needs of the Nigerian sites. An example of one lesson learned is the use of Virtual Private Network (VPN) as a solution to poor internet connectivity at one of the sites, and its adoption is underway at the other. Virtual Private Servers (VPS) or shared online hosting were also evaluated and offer alternative solutions. Installing and using REDCap in LMIC institutions for research data management is feasible; however, planning for trained personnel and addressing electrical and internet infrastructural requirements are essential to optimize its use. Building this fundamental research capacity within LMICs across Africa could substantially enhance the potential for more cross-institutional and cross-country collaboration in future research endeavors.

## 1 Introduction

While health research has increased substantially over the past decade, in LMICs and particularly Africa, it is still limited [1]. Among LMICs, countries in sub-Saharan Africa face the greatest health system gaps, further exacerbated by low health research capacity. There is a great disparity in research capacity between African countries with research in Sub-Saharan Africa accounting for only about 1% of total global research, despite being home to 15% of the world’s population [2]. A key limitation remains high quality and reliable collection, storage, and management of research data [3]. A recent paper [4] explored commonly-used research data spreadsheets in LMICs such as Microsoft Excel®, Libre Office®, Open Office®, and Google sheets®. However, Microsoft Access® and the Research Electronic Data Capture (REDCap®) systems were noted to have comparatively more functionality.

REDCap is a secure, web-based application for building and managing online surveys and databases. It was developed in the United States at Vanderbilt University in 2004 for academic and non-profit use in health research, quality assurance, and health service management [5] The original application was designed to support a small group of clinical researchers who needed a secure data collection tool that met Health Insurance Portability and Accountability Act (HIPAA) compliance standards. Based on the belief that researchers would be in the best position to take control and better develop their research if they could independently and securely manage their data. Therefore, REDCap was developed as a tool that could be used without extensive training. The system allows researchers to manage their own projects directly, including accessing data through any browser on any device. Not-for-profit institutions can join the REDCap Consortium and contribute their expertise and assistance free of charge. However, REDCap is not an open-source software and requires organizations to execute a valid end-user license agreement with Vanderbilt University to join the consortium and obtain the REDCap source codes [6]. REDCap also allows researchers to choose and define their data elements, which allows it to be used for a variety of research projects including those within 21 Code of Federal Regulations (CFR) Part 11, Federal Information Security Management Act (FISMA), and the Health Insurance Portability and Accountability Act (HIPAA)-compliant environments [5]. Table 1 shows the key features of REDCap [7].

As of December 16, 2020, REDCap is being used at 4,705 institutions in 139 countries around the world and growing according to the tracker at the top of the REDCap webpage [5]. Only 6.6% of active REDCap users are in Africa. [5] This study reports on the experiences, challenges, and lessons learned from installing and using the REDCap system for research data collection at three collaborating sites, to help institutions in LMICs (particularly in Africa) install and use REDCap and enhance their potential for inter-institutional and international collaboration in future research endeavors.

## 2 U54 epigenomic biomarkers of HIV-associated cancers project

REDCap was selected as the uniform data collection system for this ongoing collaborative study funded by the National Cancer Institute, National Institutes of Health (U54 CA221205). Our goal is to understand the epigenetic determinants of two common HIV-associated cancers, hepatocellular carcinoma (HCC) and cervical cancer, in Nigeria. The project seeks to collect clinical data from HIV-positive and negative patients with or without liver and cervical cancer including: patients’ socio-demographic information, clinical and health history, laboratory results, and specimen tracking status. The bioinformatics team uses the information collected in REDCap to create datasets that are used for project analysis. Northwestern University (NU) in Chicago, Illinois, USA serves as the lead institution and is joined by two Nigerian partners, the College of Medicine of the University of Lagos (CMUL) and Jos University Teaching Hospital (JUTH).

## 3 REDCap user agreement and system design

An end-user license agreement was signed between Vanderbilt University and the partner institutions (i.e., CMUL and JUTH) for each instance [8]. A locally hosted REDCap v8.8 and v7.1 was set up on the UBUNTU Open Source Mate Operating System, running on top of a HP ProLiant ML350e Gen8v2 Server and HP DL 380 G9 E5-2620 V3 6 core 2.4GHZ. The study involved two projects collecting datasets at both Nigerian sites simultaneously. The first project required the collection of data on patients with and without HIV and/or primary liver cell carcinoma. The second involved data collection on women with and without HIV and/or cervical cancer. Therefore, collaborators from each site created REDcap databases for each project with distinct data dictionaries made accessible via the GitHub platform for open-source collaboration. The repository, including the latest data dictionary versions and associated analysis scripts can be accessed at https://github.com/NUARIG/U54NigeriaCode. The sites used this resource to replicate identical REDCap databases on their local server by matching local database design to the latest version of the data dictionary on GitHub. Once a week, data from the Nigerian sites (CMUL and JUTH) were synchronized and transferred to NU as attachments via secure REDCap survey form. The data analysis teams at NU then merged the data sets to be used for statistical analysis.

## 4 Database design, data entry, quality control, installation experience and collaboration between sites

### Database design

The database for each project was designed using Institutional Review Board (IRB) approved protocols, which were updated to reflect any changes as necessary for the project. One example of a protocol change was an increase in initial volume of blood samples collected for the project 1 liver cell carcimona group. This resulted in a subsequent increase in the number of required aliquots, which we addressed using a branching logic to modify the original database to accommodate the additional aliquots using repeating instances.

### Data entry

Due to limited connectivity at the clinics, PDF version of data entry forms in REDCap were printed and used to collect clinical and laboratory data. Personnel with prior experience in computer use and data entry were identified and trained at both Nigerian sites to serve as data entry personnel, whereupon they entered the data from paper forms into web forms in REDCap in batches. Both sites used the data quality module on REDCap to conduct quality control checks primarily for missingness, field validation errors and outliers. The data from both sites (CMUL and JUTH) were exported from their respective REDCap instances and transferred to NU as an attachment to a secure REDCap survey form. The data were downloaded, reviewed for entry errors such as incorrect subject ID formatting as well as missing data and imported into mirror databases (clinical data and specimen data), created for each site in NU REDCap instance, using REDCap’s Data Import tool/R scripts. Once the database at NU was updated, the data were ingested by R scripts on the fly using the Application Program Interface (API) to access real-time data without requiring manual point-and-click downloads of data files. Reproducible pipelines were developed using the R Markdown scripts to generate data quality and enrollment reports. All scripts are available in Github.

As an example—Below is an excerpt of the script to read data from REDCap into R.
  myurl <—scan(“config/redcap_url”, what = “”)
  mytoken <—scan(“config/luthclinical_token”, what = “”)
  #The above two lines are credentials to access the data from REDCap
  mydata = REDCapR::redcap _read(
  redcap_uri = myurl,
  token = mytoken
  )$data
  mymetadata = REDCapR::redcap _metadata_read(
  redcap_uri = myurl, token = mytoken
  )$data
  # Data from REDCap is read into R using REDCapR package.
  #mydata is the fields/variable data and mymetadata is the basically the data dictionary which has descriptive details of the variables in the database.
  luth_data <—mydata # mydata is read into a dataframe which is used in further analysis.

### Data quality assurance

**Fig. 1:**
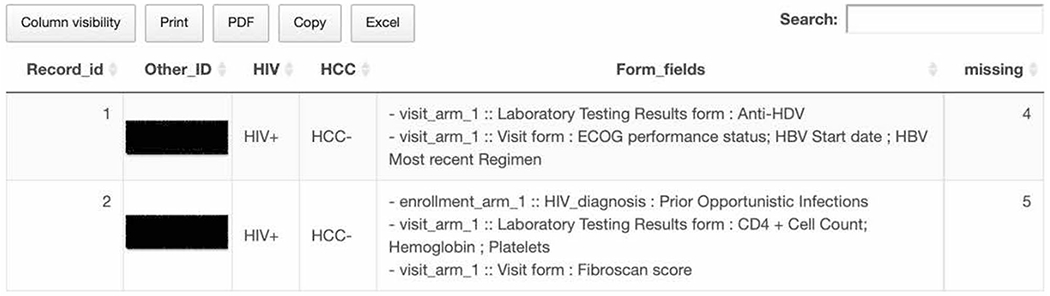
Screenshot of a data quality report

The forms in the REDCap databases which were used by the sites to enter data (in their own instances) were built with input from study clinical investigators and statisticians who are part of U54 project. They defined allowable values and field types for the case report forms. The databases were designed such that data integrity checks could occur at the point of data entry. This was achieved by determining data quality rules and allowable field values for each variable. Validation rules in REDCap were used while creating fields to improve data quality. For example, a minimum and maximum of acceptable range of values were set for some fields used to capture laboratory test results. Fields which capture dates were another example of the utility of data validation rules, as the commonly-used date format at the Nigerian sites is different from those at NU. Validation rules were applied to constrain the dates to a standardized format (yyyy-mm-dd) to avoid confusion at the time of analysis. These rules immediately flagged errors at data entry upon deviation from the accepted format. Secondary auditing of data and record locking were also utilized as measures to ensure data quality. The record-locking feature in REDCap allowed the users to prevent accidental edits to data that have already been validated. As mentioned above, both sites conducted prior quality control checks for missingness and outliers using REDCap’s data quality module to minimize missing values, field validation errors (incorrect data type, out of range), outliers for numerical fields (numbers, integers, sliders, calc fields), etc. This module allows users to conditionally execute predefined sets of data quality rules on project data to check for errors. Users can create new rules as well as edit, delete, or reorder the rules that have already been created. Users can view the details of identified errors and correct them. Data quality checks were conducted by the REDCap teams at each site and at NU. The data from two sites are merged at NU at the time of analysis and checked for other discrepancies. Figure 1 is a screenshot of a data quality report which highlights missing data in REDCap for an enrolled patient. This report was created by the NU Biomedical Informatics and Statistics Core (BISC) team using an R script utilizing API. This report complements REDCap’s internal data quality rules and site’s own data quality assurance process and act as an additional layer of data quality check. Reports were shared with data processing teams at the sites and corrections were integrated into the next upload. Once data quality issues were resolved, the record was locked to prevent further edits.

### Prior experience with database management software

**Fig. 2:**
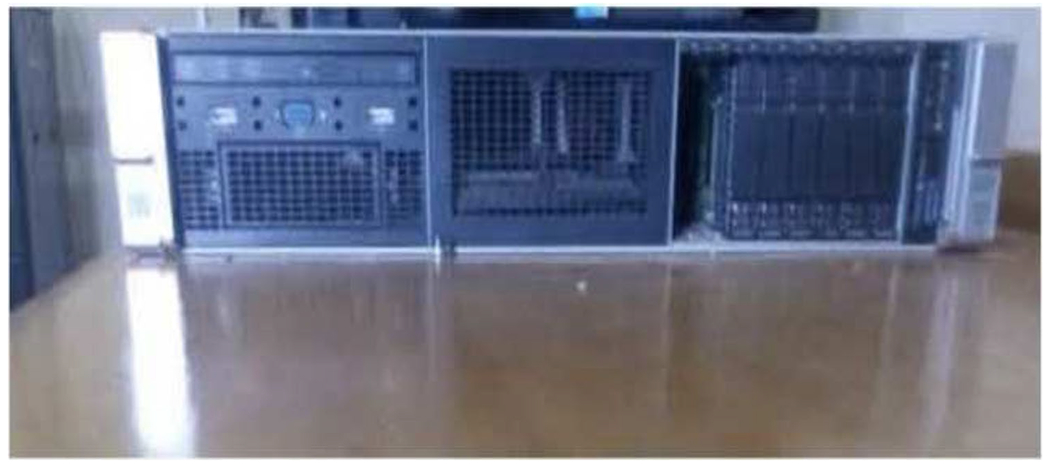
Dedicated RedCap Server at JUTH

Neither research team (CMUL or JUTH) had any experience with REDCap prior to this project. Both sites had to purchase a dedicated server preinstalled with the Ubuntu Mate Operating system in order to access REDCap as seen in Fig. 2.

### Installation experience

Navigating the actual REDCap installation was user-friendly. The data team at both Nigerian sites were mostly familiar with key REDCap installation features such as project setup, data quality, and security configuration checks. However, they had to engage in self-learning to ensure optimum REDCap functionality. For example, the server was reconfigured for optimal performance using a web server on Ubuntu, working with GitHub, and interfacing with Excel. The Nigerian teams were able to self-teach various REDCap processes by searching for videos on Google or focused virtual meetings with the NU team. Knowledge and skill sharing occurred frequently between sites.

### Reporting

The REDCap Application Program Interface (API) was used to generate automated data quality reports and descriptive statistics. Examples of R scripts are included in the public GitHub repository.

### Training and backup support

At launch, team members from NU visited both local sites and trained Nigerian team members. In addition, one team member from each Nigerian site was sponsored to attend the Annual REDCap Conference. Weekly or bi-weekly meetings were scheduled with the Nigerian and NU teams to identify challenges, troubleshoot, and address any questions or problems encountered by either site. In addition, NU provided periodic hands-on training and REDCap support for the Nigerian the teams. This support ranged from troubleshooting issues with data entry to tips on how to use advanced Excel features that interfaced with the database (pivot tables, excel functions, formatting of data, etc.) for cleaning and analyzing data exported from REDCap.

### Impact on patient care and research

REDCap has enabled us to conduct data collection, management, sharing, and analysis with rigorous electronic security in a very cost and time efficient manner, facilitating both our ongoing research collaboration and those still to come. This has proven particularly important during the travel restrictions imposed in response to the COVID19 pandemic, which limited the abilty for the NU team to be on-site in Nigeria for both hands-on training and joint data quality checks. The flexibility and customizability of the system, all while maintaining data security, have allowed these data to be available for clinical use where appropriate. Thus, REDCap has also made substantial contributions to patient care, in particular HIV-infected cancer patients. The demographic, epidemiologic, and clinical data accessible in REDCap have helped physicians provide more personalized care, and has even facilitated greater involvement by patients in their own health care.

The REDCap system has greatly enhanced local research capacitiy in Nigeria by allowing us to study the epigenetic biomarkers in relations to under-studied patient factors including viral infection, demographics, and risk factor exposures. It also provides substantial opportunities for the next generation of scientists to explore and apply their own ideas and conduct their own research projects. Thus, we anticipate that these clinical and research applications of REDCap are just the foundation. Our team plans to add more detailed data on important variables such as family history, patient treatment history, and lifestyle factors (diet, smoking, etc.). As additional patient data and researcher interest accrue around these projects, both the scope of the REDCap database implemented in Nigeria and its impact on both patient care and health research could expand exponentially.

Already, five abstracts have been presented at the NCI’s 9th Annual Symposium on Global Cancer Research (ASGCR) and the 2021 American Society of Clinical Oncology meeting. These projects used our data from REDCap to analyze factors such as epigenetic age, biomarkers, coinfection rate with cancer and HIV, and clinical characteristics. One abstract presented at ASGCR, “Characteristics of Hepatocellular Carcinoma in Nigerians with and without HIV,” was selected to be published in a special issue of *Cancer Epidemiology, Biomarkers, & Prevention* that highlights work from the meeting.

## 5 Challenges with REDCap installation and use

### REDCap and server installation

Prior experience with server installation was not uniform and varied between sites. Personnel without the prerequisite skills had to be trained in system administration.

### Availability of continuous electrical power

Initially, the server was powered by a local electrical supply, which failed sporadically for various periods of time. This was a major challenge at both sites. While each institution has a back-up generator that helped ease this challenge, it proved inadequate to meet the needs required for the project’s data entry and data quality check procedures. This challenge was successfully overcome by installing a Schneider Electric 3KVA APC UPS, at a cost of ~ $1,000, which provides high-density, true double-conversion online power protection for servers that lasts for up to 5 h and was sufficient to run the REDCap server for local data entry at the Nigerian sites.

### Internet provision, data connectivity, and data speed

Each site selected the most reliable local internet service available. To reduce internet service costs, the server was not connected to the internet at all times. The local area network was gated to the public internet only at times when data transmission was needed.

### Remote access to the REDCap database

Patient data were collected at locations far from the REDcap server, requiring remote access for data entry. Initially, data collectors used a local internet server to access the database directly, but interrupted internet connectivity and poor data speed interfered considerably with this process. To address this challenge, a Virtual Private Network (VPN) was established at JUTH and solved the issues. A similar VPN is in the process of being established at CMUL.

### Importing REDCap data dictionaries via GitHub

Initially, there were challenges configuring each project’s database at each site, when downloading each data dictionary from Github (https://github.com/NUARIG/U54NigeriaCode). For instance, when attempting to import a data dictionary, REDCap tended to time out. Access rights privileges caused these problems which were addressed by “forking” the files into a GitHub repository prior to importing them.

### Lack of trained and experienced personnel

As this was the first time that REDCap was introduced at each Nigerian institution, few personnel had any prior experience with it,as with many LMICs. Members of the REDCap team from both JUTH and CMUL attended the REDCap Conference 2018 in Chicago, an interactive workshop primarily geared towards REDCap administrators. Attending the REDCap conference gave the team members not only a basic working knowledge of REDCap but also provided an opportunity to get hands-on experience in using the available components creatively to support customized pipelines. They also attended a workshop held by the NU data team to go over the project specific setup for U54 REDCap databases. Finally, the Nigerian team members’ also relied upon their membership in the REDCap consortium to provide a means with which to interact with fellow REDCap user community members around the world.

### Location of the server

The Nigerian sites experienced some challenges in identifying an appropriate location for our servers. The location had to balance safety/security of the server with access to a stable internet connection. To ensure the safety and security of the data and to reduce data consumption, connection to the internet occured only during data uploads and/or transfers.

### Updating REDCap

Updating REDCap to its most recent version was a constant challenge, as poor internet connectivity and speed impeded REDCap servers’ direct communication with the REDCap consortium server to download the updates. Additionally, REDCap’s Easy Upgrade feature cannot be used when the REDCap web server is using multiple application servers. Therefore, the Nigerian teams had to perform manual upgrades using the SQL Upgrade Script. It is believed that a Virtual Private Server (VPS) web host or VPN installation would solve the problem, but have yet to test that solution in practice.

### Report generation

Generating reports required an understanding of database query logic which was not always self-evident. When extracting data from the database to support articles/abstracts/analyses, the report created using REDCap’s Report module had to accurately represent the requisite query criteria and exported into a.CSV file for analysis.

Table 2 summarizes the main challenges of the REDCap installation and our resolution.

## 6 Lessons learned

### Value of teamwork and regular communication between sites

Teamwork was extremely important to the success of installing and using REDCap. Regularly scheduled communications between the teams at all three sites (NU, CMUL, and JUTH) via BlueJeans/Zoom occurred at least bi-weekly. This involved discussions around the initial project setup, data quality checks, and verification of data collection protocols with the clinical teams. The NU team was always available to provide clarifications on any aspect related to REDCap and project associated data. Additional ad hoc calls were set up on Google hangouts or Zoom as needed. There were also regular meetings and transfer of knowledge and skills between the Nigerian sites. For instance, both Nigerian sites directly shared their REDCap database templates and structures. The mode of communication between the Nigerian sites was primarily by cellular or WhatsApp text or voice calls as well as emails. One challenge was that the Nigerian and US teams are located in different time zones (5 or 6-h time difference depending on the time of year) causing difficulty in scheduling mutually convenient meeting times.

### Need for locally contextualized processes

Within the limited resources available to run a sophisticated REDCap infrastructure, the Nigerian teams were able to replicate a workable REDCap database locally. The power supply solution and operational configuration for intermittent, deliberate access mitigated the risks and resource constraints associated with supporting a web server for 24 h a day.

### Sustainability of REDCap beyond the life of the U54 project

The need for local investments in a VPS or VPN would enable remote access and ensure a larger network of REDCap users within the academic and clinical communities. The local Nigerian sites were able to use the knowledge and skills garnered from this project to successfully create new REDCap databases for some of the small seed awards given by the primary U54 grant to Nigerian awardees. These also include periodic data quality checks and technical support provided as needed, similar to that described above, illustrating the expanding impact of the REDCap system on continuing to develop local Nigerian research projects.

## 7 Recommendations for future developments

We are hopeful that as we continue to use REDCap, more researchers with similar interests will have the opportunity to benefit from its capabilities and consider using it for their research projects. Looking forward, we recommend that other LMIC institutions consider investing resources to build the capacity to facilitate their researchers’ adoption of REDCap. This includes plans to address the needs associated with scaling-up local servers to an online web-based server for entire institutions and possibly local researchers. We look forward to using REDCap mobile apps for easier offline data capture at the point of care and at remote data collection sites.

## 8 Conclusion

We described the experiences, challenges, and lessons learned from installing and using REDCap, a system that is free to use and does not require a dedicated IT team, in Nigeria. We found REDCap to be a powerful and user-friendly tool for collaborative research across multiple sites. The main challenges resulted from unstable power supply, intermittent reliable internet access, and the need to fit REDCap into our local settings. We learned that teamwork, on-the-job training, and effective communication across sites helped us navigate some of these challenges. The infrastructure that we established will serve as a launching pad for other projects at our institutions, with lowering future overhead costs and facilitating future collaborations. We believe that our approach will be useful to institutions in LMIC settings that face similar challenges; therefore we published the artifacts (instruments, code) that resulted from this project online at (https://github.com/NUARIG/U54NigeriaCode). We urge colleagues at institutions in LMIC settings to utilize and build upon these tools when conducting multisite collaborative research. We look forward to receiving feedback on these tools and to collaborating in the construction of reproducible methodologies and open science feasible for deployment to LMICs.

## Figures and Tables

**Table 1:** REDCap Key Features

Online or offline project design	Can be used online using the Online Designer or offline using a “data dictionary” template in Microsoft Excel that can be uploaded into REDCap
**Availability**	Software is available at no cost for REDCap Consortium Partners
**Secure and web-based**	Data can be directly entered via a web interface directly or via an online survey. The database “project” can be built from anywhere in the world over a secure web connection with authentication and data logging
**A quick and easy software installation process**	Fast and flexible; the entire process from conception to production-level database or surveys can take less than one day
**Multi-site access**	Databases/surveys can be used by researchers from multiple sites and institutions across countries, regions, and continents
**Autonomous utilization**	Research groups have complete autonomy and control to add new users
**Fully customizable**	Fully customizable; database/surveys can be designed as the researcher deems fit
**Audit trails**	Audit trails serve as a useful quality control function for tracking data manipulations and user activity
**Automated export procedures**	Permits seamless data downloads to Excel, PDF, and common statistical packages (SPSS, SAS, Stata, R)
**A built-in project calendar**	Ensures timely tracking of REDCap activity
**A scheduling module**	Activities can be scheduled
**Regulatory compliance**	Can be installed in a variety of environments for compliance with such standards as HIPAA, 21 CFR Part 11, FISMA (low, moderate, high), and international standards. REDCap is fully personalized to meet specified user security policies
**Available in multiple languages**	Several language translations have been compiled (e.g. Chinese, French, German, Portuguese), and it is anticipated that other languages will be available in full versions of REDCap soon
**Trial demonstrations**	Offers a one-week trial demonstration to familiarize users with its interface and functionalities
**REDCap mobile app for IOS and Android**	Mobile app allows for flexible data entry particularly in remote locations
**Other advanced features**	Features, such as ad-hoc reporting tools, branching logic, file uploading, calculated fields, and remote programmatic access via Application Programming Interfaces (API) are available

**Table 2:** Key REDCap Challenges and Resolution

	REDCap Challenges	Our resolution
1	REDCap server setup and installation technical know-how	Training and retraining Regular meetings between the sites
2	Lack of regular electricity	Institutional backup power supply Installation of 3KVA APC UPS
3	Internet service provision, data connectivity and speed	Pre-identify internet service which works best at server location Periodic connections of the server to reduce internet costs
4	Difficulty in data entry at remote sites	VPN connectivity obtained at JUTH, plans underway to replicate at CMUL
5	Importing REDCap data dictionaries via GitHub, problems of access privileges	Files forked into another GitHub account and then re-downloaded
6	Lack of trained and experienced personnel	Training and retraining Regular meetings between the sites Team work
7	Siting of the local server	Balance between feasibility, safety and security Explored options for remote access using an online shared hosting platform
8	Upgrading REDCap: Server could not communicate with the REDCap consortium server directly	Manual upgrade using SQL Upgrade Script VPS or VPN installation might solve this problem

## References

[1] Morel T, Maher D, Nyirenda T, Olesen OF Strengthening health research capacity in sub-Saharan Africa: mapping the 2012–2017 landscape of externally funded international postgraduate training at institutions in the region. Global Health. 2018;14(1):77. 10.1186/s12992-018-0395-0 30064479 PMC6066939

[2] Uthman OA, Wiysonge CS, Ota MO, Increasing the value of health research in the WHO African Region beyond 2015-reflecting on the past, celebrating the present and building the future: a bibliometric analysis. BMJ Open. 2015;5(3):e006340. 10.1136/bmjopen-2014-006340 PMC4360830 25770227

[3] Missinou MA, Olola CH, Issifou S, Short report: Piloting paperless data entry for clinical research in Africa. Am J Trop Med Hyg. 2005;72(3):301–3. 15772326

[4] Taylor DM, Hodkinson PW, Khan AS, Simon EL Research skills and the data spreadsheet: A research primer for low- and middle-income countries. Afr J Emerg Med. 2020;10(Suppl 2):S140–4. 10.1016/j.afjem.2020.05.003. 33304797 PMC7718460

[5] REDCap: About. Vanderbilt. Accessed October, 2020. https://projectredcap.org/about/

[6] Harris PA, Taylor R, Minor BL, The REDCap consortium: Building an international community of software platform partners. J Biomed Inform. 2019:95: 103208. 10.1016/j.jbi.2019.103208. 31078660 PMC7254481

[7] REDCap: Software. Vanderbilt. Accessed October, 2020. https://projectredcap.org/software/

[8] REDCap: REDCap License Terms. Vanderbilt. Accessed October, 2020. https://projectredcap.org/partners/termsofuse/

